# Endovascular Therapy for Concurrent Cardio-Cerebral Infarction in a Patient With Trousseau Syndrome

**DOI:** 10.3389/fneur.2019.00965

**Published:** 2019-09-06

**Authors:** Kenichi Sakuta, Taiji Mukai, Asako Fujii, Kentaro Makita, Hiroshi Yaguchi

**Affiliations:** ^1^Department of Neurology, The Jikei University School of Medicine, Kashiwa Hospital, Kashiwa, Japan; ^2^Department of Cardiology, The Jikei University School of Medicine, Kashiwa Hospital, Kashiwa, Japan

**Keywords:** cardiocerebral infarction, Trousseau syndrome, endovascular therapy, myocardial infarction, ischemic stroke

## Abstract

Only a few patients have been reported to undergo endovascular therapy for Trousseau syndrome. This is the first report of a patient with Trousseau syndrome who developed synchronous cardiocerebral infarction and underwent endovascular therapy for both. A 55-year-old woman with Trousseau syndrome arising from stage IV ovarian cancer presented with consciousness disturbance, aphasia, and right hemiparesis. Magnetic resonance imaging showed acute cerebral infarction limited to the left basal ganglia and occlusion of the left middle cerebral artery (MCA). Electrocardiography showed ST elevation in leads II, III, and aVF with reciprocal change. Mild elevation of myocardial enzymes was observed in laboratory data. She was diagnosed with synchronous cardiocerebral infarction. Both infarctions were considered as appropriately indicated for endovascular therapy. Since her vital signs were stable, a decision was made to treat the cerebral infarction first. Thrombectomy with a stent retriever was performed, which achieved complete recanalization of the left MCA. Percutaneous coronary intervention successfully recanalized the occluded right coronary artery. She suffered no recurrence of stroke or acute coronary syndrome upon heparin administration. Cardiocerebral infarction caused by Trousseau syndrome is rare and demands optimal planning of endovascular therapy.

## Introduction

Trousseau syndrome is a condition characterized as hypercoagulation disorder related to malignancy (1). It is considered a common cause of ischemic stroke in terminally ill patients, and several affected patients have been reported to undergo mechanical thrombectomy (2–4). Here, we report a rare case of synchronous cardiocerebral infarction (CCI) caused by Trousseau syndrome in a patient who underwent endovascular therapy to treat both infarctions.

## Case Report

**Figure 1:**
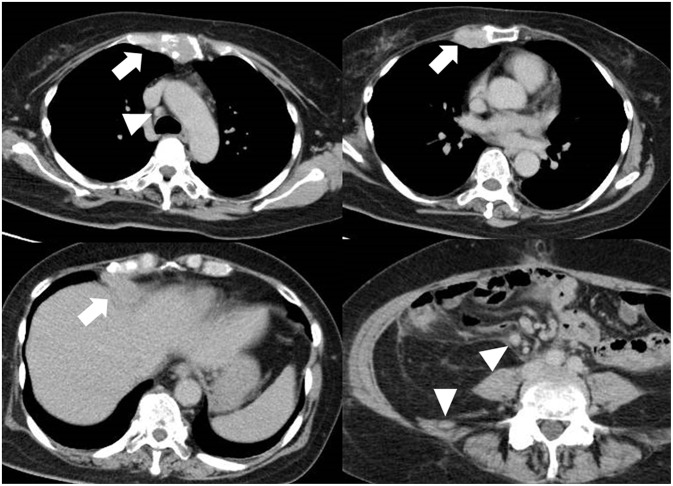
Multiple metastasis. Although the ovary carcinoma was resected, liver, and bone metastasis (arrow) was detected. Multiple lymph nodes are swelling (arrowhead), indicating metastasis.

**Figure 2:**
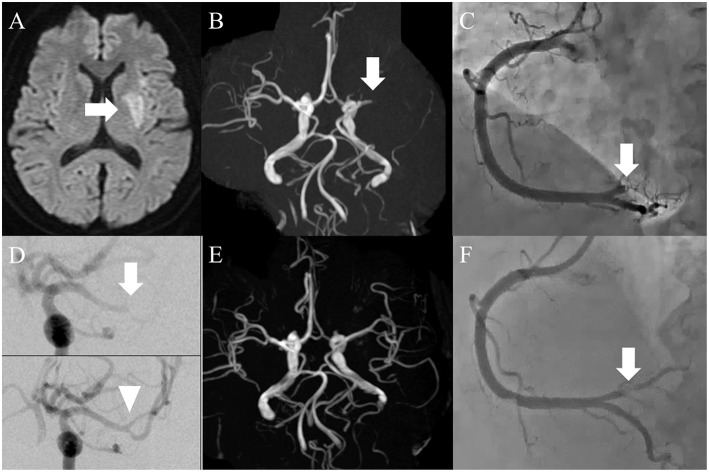
**(A)** Magnetic resonance imaging on admission day. Diffusion-weighted image showed acute infarction limited to the left basal ganglia (arrow). **(B)** Magnetic resonance angiography. The left middle cerebral artery (MCA) was occluded in the proximal portion (arrow). **(C)** Coronary angiography on admission day. The distal portion of the right coronary artery was completely occluded (arrow). **(D)** Thrombectomy for cerebral artery occlusion (arrow), whereby complete recanalization was achieved (arrowhead). **(E)** Brain MRA on the second hospital day. The left MCA was recanalized successfully. **(F)** Coronary angiography post-intervention. Although the stenosis remained (arrow), recanalization was achieved.

A 55-year-old woman with a history of ovarian carcinoma and multiple metastasis to lymph node, liver, and bone (Figure 1) was admitted to our hospital with consciousness disturbance. Three months prior to admission, she had suffered deep vein thrombosis and pulmonary embolism attributable to Trousseau syndrome and was prescribed direct oral anticoagulants (DOACs). She was expected to survive at least 6 months if chemotherapy could be continued, and Karnofsky Performance Status (KPS) was 90. On examination, her Glasgow Coma Scale score was 10 (E3, V1, M6), body temperature 36.0°C, blood pressure 133/78 mmHg, and heart rate 100 beats/min. Neurological examination revealed motor aphasia, right hemiparesis, and conjugate deviation of eyes to the left. The National Institutes of Health Stroke Scale score was 23. She reported no chest pain. Blood tests showed mild elevation of myocardial marker enzymes such as aspartate aminotransferase (45 U/L), lactate dehydrogenase (425 U/L), creatine kinase (777 U/L), creatine kinase MB (35 U/L), and troponin T (0.21 ng/mL; normal, <0.05 ng/mL). Tumor makers were elevated, especially CA-125 (190 U/mL; normal, <35 U/mL), and D-dimer was 11.4 μg/mL on admission, which gradually increased despite DOAC continuation. We performed brain magnetic resonance (MR) imaging and MR angiography immediately after admission. Acute cerebral infarction was detected in the left basal ganglia, and the left middle cerebral artery (MCA) was occluded (Figures 2A,B). Electrocardiography on admission revealed normal sinus rhythm with ST-segment elevation in leads II, III, and aVF, with reciprocal change in leads aVL and V2–V6. Bedside transthoracic echocardiography revealed almost normal wall motion without evidence of intracardiac thrombus, aortic dissection, or vegetation. These results led us to the diagnosis of synchronous CCI.

Both infarcts were considered suitably indicated for endovascular therapy, although tissue plasminogen activator was a contraindication because the time of onset was unknown. Since the vital signs were relatively stable, a decision was made to treat the MCA occlusion before the coronary artery occlusion. A cerebral angiogram showed distal MCA occlusion (Figure 2D, arrow), and the thrombus was successfully retrieved with a stent retriever (Figure 2D, arrowhead). The white-colored thrombus was collected. Since the awareness of the patient was delayed, time from last known well to recanalization was 19 h. Percutaneous coronary intervention (PCI) was subsequently performed using the same femoral sheath. A coronary angiogram showed complete occlusion of the distal right coronary artery at the atrioventricular branch (Figure 2C), and after plain old balloon angioplasty the occluded artery was recanalized (Figure 2F). After the intervention, ST elevation on electrocardiography was normalized. Since treatment with heparin injection for secondary stroke prevention was scheduled, stent placement was not performed as this requires dual-antiplatelet therapy, raising the fear of bleeding complications. After the procedures the patient was started on heparin injection instead of DOACs. Brain MRA on the second hospital day showed a recanalized left MCA (Figure 2E). The patient achieved a functional status of modified Rankin score 3 and KPS 70 at 3-month follow-up with no recurrence of infarctions under continuation of heparin subcutaneous injection. Such a good recovery made it possible to restart her chemotherapy.

## Discussion

The unique point of the present case is that simultaneous CCI occurred in a patient with Trousseau syndrome. Furthermore, both occluded arteries were successfully recanalized with endovascular therapy.

This is the first report of dual endovascular intervention in a CCI patient with Trousseau syndrome. Endovascular therapy for cerebral infarction in Trousseau syndrome is rarely reported (2–4). All of these previous cases exhibited terminal stage of cancer, elevated D-dimer, and thrombus with white coloration consisting of fibrin and platelets. The present case is consistent with these characteristics. In cases where the prognosis is unfavorable because of terminal-stage cancer (3, 4), it is sometimes difficult to make a decision to undertake invasive surgery in patients in such poor condition. Recent article regarding endovascular therapy for cancer-related stroke revealed that the rate of good outcome at discharge was not significantly differ compared to those without cancer (5). In the present case we readily decided to perform surgery because the patient was expected to survive another 6 months in the absence of infarction.

Acute CCI is defined as synchronous infarction of the brain and heart, and is a life-threatening condition that requires immediate medical intervention (6). Frequency is reported as 1.6% in a cohort of acute ischemic stroke patients and 0.9% in a cohort of acute coronary syndrome patients (7, 8). In the case of CCI with indications for both endovascular therapies, no evidence-based treatment plan has been established. Some authors recommend administration of intravenous tissue plasminogen activator of stroke dose, followed by PCI (6, 9). Thrombectomy for cerebral infarction takes precedence over PCI only when there is basilar occlusion or non-ST elevation myocardial infarction (9). In the present case cerebral infarction of the anterior circulation was treated first, followed by the ST-elevation myocardial infarction. The reason why the outcome was favorable in this case may be because hemodynamics were maintained and arrhythmia did not develop despite the inferior wall infarction.

## Conclusion

Trousseau syndrome rarely causes major vessel occlusion and in the case of CCI, hemodynamics stability may play an important role in determining the appropriate ordering of treatment.

## Data Availability

All data containing relevant information to support the study findings are included in the manuscript.

## Ethics Statement

The authors declare that ethics approval was not required for this case report. Written informed consent was obtained from the patient for publication of this case report and any accompanying data.

## Author Contributions

KS drafted the manuscript and prepared the figures. TM designed the study and analyzed the clinical data. AF collected the clinical data and interpreted the data. KM collected the clinical data and interpreted the data. HY helped to write and revise the manuscript. All authors have read and approved the final manuscript.

### Conflict of Interest Statement

The authors declare that the research was conducted in the absence of any commercial or financial relationships that could be construed as a potential conflict of interest.

## Abbreviations

CCI: cardiocerebral infarction
DOAC: direct oral anticoagulant
MCA: middle cerebral artery
MRA: magnetic resonance angiography
PCI: percutaneous coronary intervention.
